# Free Gingival Graft to Increase Keratinized Mucosa after Placing of Mandibular Fixed Implant-Supported Prosthesis

**DOI:** 10.1155/2017/5796768

**Published:** 2017-02-15

**Authors:** Danny Omar Mendoza Marin, Andressa Rosa Perin Leite, Lélis Gustavo Nícoli, Claudio Marcantonio, Marco Antonio Compagnoni, Elcio Marcantonio

**Affiliations:** ^1^Araraquara Dental School, Department of Dental Materials and Prosthodontics, Universidade Estadual Paulista (UNESP), Araraquara, SP, Brazil; ^2^Araraquara Dental School, Department of Diagnosis and Surgery, Universidade Estadual Paulista (UNESP), Araraquara, SP, Brazil; ^3^Araraquara Dental School, Department of Postgraduate Studies in Implantology, University Center of Araraquara (UNIARA), Araraquara, SP, Brazil

## Abstract

Insufficiently keratinized tissue can be increased surgically by free gingival grafting. The presence or reconstruction of keratinized mucosa around the implant can facilitate restorative procedure and allow the maintenance of an oral hygiene routine without irritation or discomfort to the patient. The aim of this clinical case report is to describe an oral rehabilitation procedure of an edentulous patient with absence of keratinized mucosa in the interforaminal area, using a free gingival graft associated with a mandibular fixed implant-supported prosthesis. The treatment included the manufacturing of a maxillary complete denture and a mandibular fixed implant-supported prosthesis followed by a free gingival graft to increase the width of the mandibular keratinized mucosa. Free gingival graft was obtained from the palate and grafted on the buccal side of interforaminal area. The follow-up of 02 and 12 months after mucogingival surgery showed that the free gingival graft promoted peri-implant health, hygiene, and patient comfort.* Clinical Significance*. The free gingival graft is an effective treatment in increasing the width of mandibular keratinized mucosa on the buccal side of the interforaminal area and provided an improvement in maintaining the health of peri-implant tissues which allows for better oral hygiene.

## 1. Introduction

Fixed implant-supported prosthesis is an alternative treatment in prosthodontics mandibular rehabilitation [1]. However, the maintenance and health of the peri-implant soft tissue is necessary for the longevity of dental implants [2] and prosthesis. The soft tissue healing following implant surgery may result in the establishment of a border tissue composed of either keratinized or nonkeratinized mucosa [3].

A study showed that an amount ≥2 mm of keratinized mucosa (KM) is needed to maintain the health of periodontal tissues providing a soft tissue seal around natural teeth [4]. However, peri-implant health with presence or absence of a minimal zone of keratinized tissue around dental implants has been studied and the literature showed divergent theories [5]. A literature review showed no significant association between “inadequate” keratinized tissue with higher plaque scores and mucosal inflammation [3]. Other studies showed that absence of adequate KM around dental implants is associated with increased plaque accumulation, mucosal inflammation, mucosal recession, and attachment loss [6, 7]. Furthermore, patient discomfort when performing oral hygiene was reported to be painful as a result of KM absence surrounding the implant, as well as mechanical irritation due to the mobility of the nonkeratinized tissue under function [3, 8, 9].

The weak sealing ability of the peri-implant nonkeratinized tissue [10], the critical bacterial plaque control in some patients [7], pain, and discomfort are the main reasons for justifying a gingival graft on the implant site [11] with absence of KM using a mandibular fixed implant. Thus, the aim of this clinical case report is to describe an oral rehabilitation procedure of an edentulous patient with absence of KM in the interforaminal area, using a free gingival graft associated with a mandibular fixed implant-supported prosthesis.

## 2. Case Description

**Figure 1:**
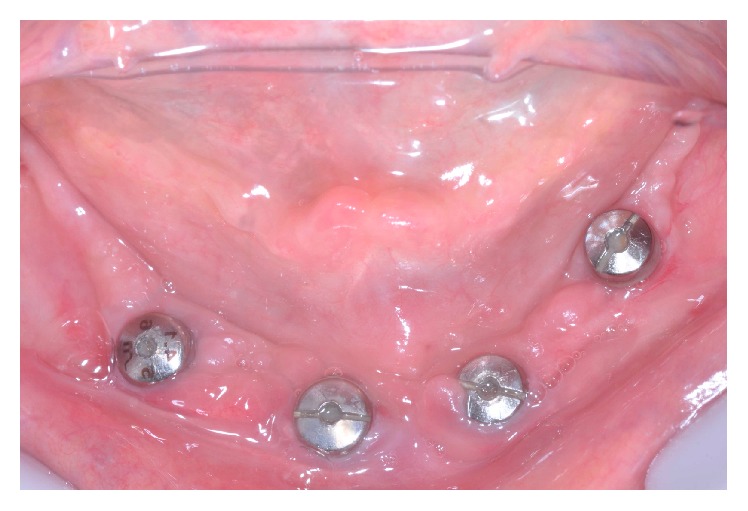
Initial appearance of the gingival tissue around the implants.

A 60-year-old, nonsmoking, female patient in good general health came to the Department of Dental Materials and Prosthodontics at Araraquara Dental School complaining that her maxillary complete denture was unstable. Clinical and radiographic examinations revealed an old maxillary complete denture and four osseointegrated dental implants in the interforaminal area with their healing caps. In addition, it was verified on the mandible the absence of KM on the buccal side of interforaminal area, shallow vestibule, presence of bacterial place around the healing caps, and complaint of painful symptoms in the gingival tissue around the implants (Figure 1). In addition, the manufacturing of a mandibular fixed implant-supported prosthesis was also needed.

Considering the patient's age, health, and comfort, as a first step, the proposed treatment was the manufacturing of a new conventional maxillary complete denture and a mandibular fixed implant-supported prosthesis. After 30 days, as a second step, a free gingival graft associated with the mandibular fixed implant-supported prosthesis was indicated for maintenance of peri-implant tissue after 30 days of installation of the new prostheses because the patient had pain and difficulty during hygienization of the mandibular prosthesis and presence of plaque accumulation.

**Figure 2:**
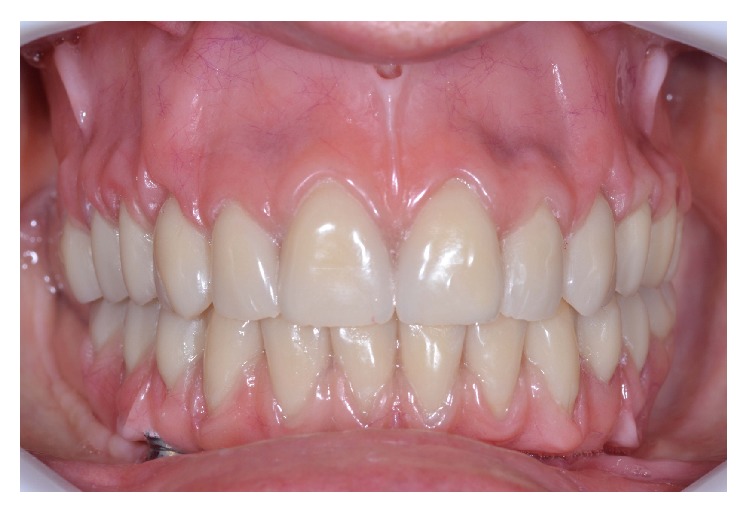
Checking the fitting of prostheses in mouth.

All procedures for manufacturing of a new conventional maxillary complete denture in combination with a mandibular fixed implant-supported prosthesis were used. A record base with an occlusion rim was used to reestablish occlusal planes and the occlusal vertical dimension and record patient's centric relation. Afterwards, the definitive casts were mounted in a semiadjustable articulator and artificial acrylic teeth were set and after evaluated in the patient. A mandibular multifunctional guide was manufactured for definitive impression and occlusal registration. Four miniabutments (Micro Unit Abutment, Conexão Sistemas de Prótese, São Paulo, Brazil) were installed on the implants and the impression was performed using impression coping (Impression Coping Micro Unit Abutment, Conexão Sistemas de Prótese, São Paulo, Brazil) and occlusal records were performed on the multifunctional guide. Heat-polymerized polymethyl methacrylate resin (Lucitone 550, Dentsply International Inc., New York, USA) was used for manufacturing the maxillary complete denture and mandibular fixed implant-supported prosthesis. Afterwards, both prostheses were installed and the fitting and adaptation were checked. (Figure 2).

**Figure 3:**
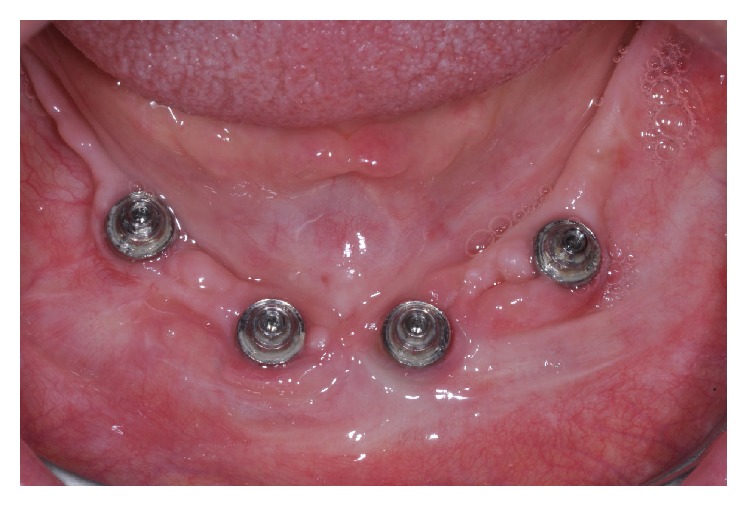
Plaque accumulation on the prosthetic components after 30 days of installation of definitive prostheses.

**Figure 4:**
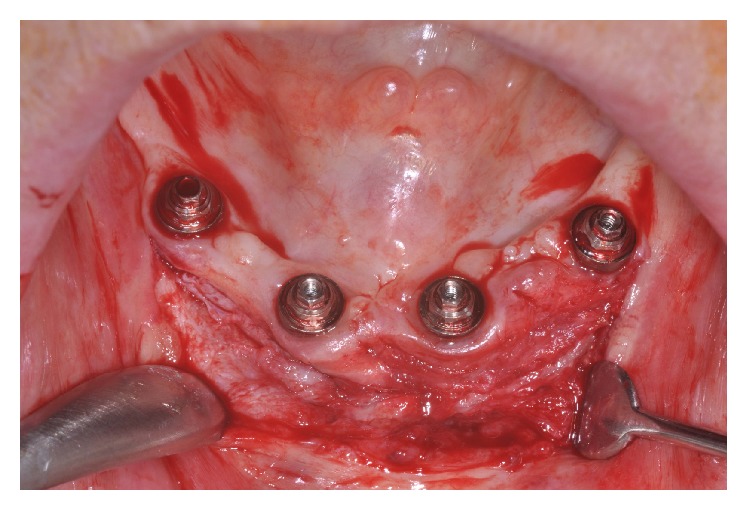
Partial-thickness flap on the buccal side of the interforaminal area around the implants.

**Figure 5:**
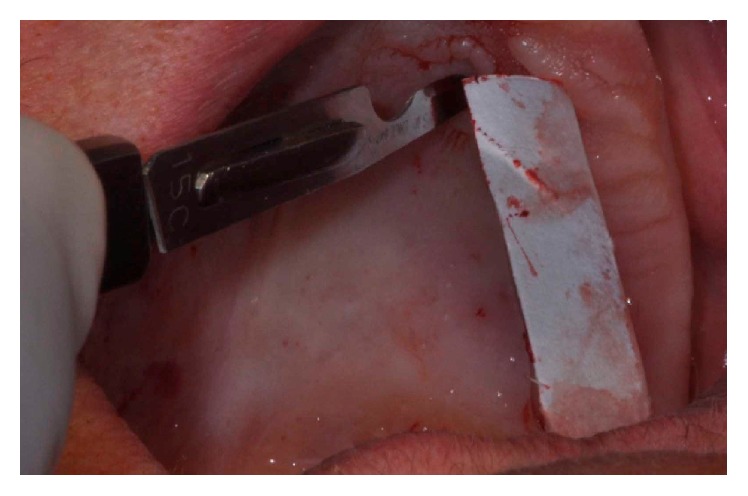
A sterile paper to make a map with the size of the recipient bed and transferred to the palate.

After 30 days, the patient returned for maintenance of the prostheses and plaque accumulation was observed in the peri-implant area on the prosthetic components (Figure 3). In addition, pain and difficulty during hygienization of the mandibular prosthesis were verified. Therefore, a free gingival graft surgery was performed to provide a KM in the peri-implant area, thus, minimizing the sensitivity during hygiene. The patient was anesthetized locally with mepivacaine 2% associated with epinephrine 1 : 100,000 (Mepiadre-New DFL Ind. e Com. S.A., Rio de Janeiro, Brazil). An intrasulcular incision was performed and a partial-thickness flap was made on the buccal side of the interforaminal area around the four dental implants (Figure 4). A sterile paper was used to make a template with the same size of the recipient bed, which was transferred to the palate in order to remove two free 1.5 mm thick gingival grafts (Figure 5).

**Figure 6:**
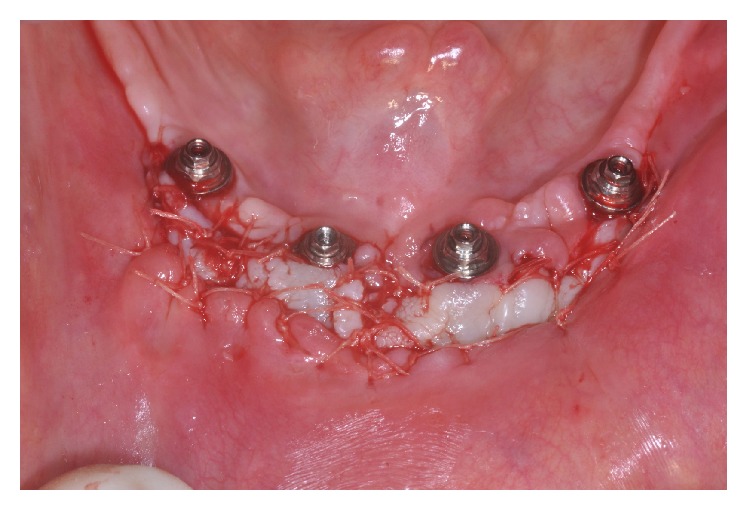
Free gingival graft placed around the implants.

A free gingival graft [12, 13] was obtained from the right and left side portions of the palate, approximately 2 mm below the gingival margin. One portion of the graft was placed covering the left surgical area and fixed by compression sutures using absorbable thread (Vicryl–Ethicon, Johnson & Johnson do Brasil, São José dos Campos, Brazil) to remain stable and in close contact with the periosteal bed. The same protocol described above was applied to the second portion of the graft on the right surgical area (Figure 6).

**Figure 7:**
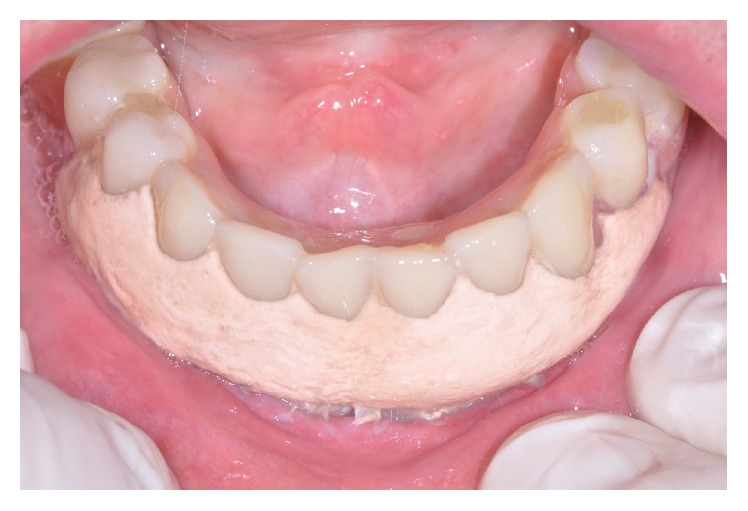
Surgical cement in the recipient bed and stabilized on the mandibular prosthesis.

The palatal donor sites were sutured using 4-0 silk threads (Ethichon–Johnson & Johnson Medical Limited, New Brunswick, NJ) to promote hemostasis and clot stabilization. Following, surgical cement (Coe-Pack-GC Europe N.V.) was added onto the palatal donor sites along with the new maxillary complete denture to aid in healing by second intention and provide comfort to the patient during the postoperative period. The mandibular fixed implant-supported prosthesis was installed in the mouth and surgical cement was added in the recipient bed and stabilized on the mandibular prosthesis (Figure 7).

Postoperative care included a 0.12% chlorhexidine rinse twice daily for 2 weeks, 500 mg of amoxicillin 3 times a day for 7 days, 100 mg of nimesulide 2 times a day for 3 days, and 500 mg of paracetamol as needed for pain. Surgical cement was replaced in the conventional maxillary denture after 48 h and 7 days, respectively, to avoid food impact between the prosthesis and mucosa. In mandible, surgical cement was replaced after 7 days after the surgical procedures. The surgical cement was removed completely in both prostheses after 14 days. Sutures of donor and recipient sites were removed after 14 days and healing took place without postoperative discomfort to the patient. Patient was recalled after 1, 3, and 6 months for follow-up, when instructions regarding home oral hygiene techniques were reinforced.

**Figure 8:**
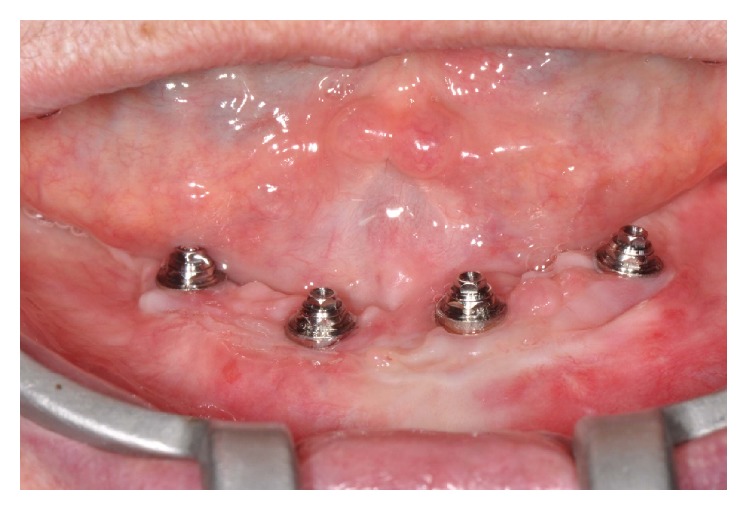
Six months of follow-up after surgery showed a good health of peri-implant tissues and absence of plaque accumulation.

After a 6-month period of follow-up (Figure 8), it was observed an improvement of thickness and a 3 mm increase in the height of keratinized mucosa, promoting a good peri-implant health and facilitating the hygiene procedures. After one year of follow-up, the patient reported being satisfied with the treatment and an improvement in the ease of cleaning the mandibular prosthesis without any complaint of painful symptoms in the peri-implant area.

## 3. Discussion

The absence of KM, around the peri-implant tissue [14], could lead to an inadequate oral hygiene, plaque accumulation, mucosal inflammation, bleeding on probing, mucosal recession, and alveolar bone loss that could negatively influence the long-term maintenance of dental implants and prosthesis [9, 15]. Several surgical procedures have been used to increase KM around implant including free gingival grafts, connective tissue grafts, pedicle grafts, and apically positioned flaps [16–18].

Free gingival graft is a successful and predictable technique [19] that could prevent hard and soft tissue problems developed after implant rehabilitation [20]. This procedure can be performed previous to implant placement, during the second stage surgery in implants or after placing of the final prosthesis [19]. Free gingival graft previous to implant placement or during the second stage surgery can result in a greater waiting time for realization of rehabilitation treatment [21]. The patient cannot wear this prosthesis during healing graft period and this could have an impact on their physiological functions, especially in patients who suffer from pain and discomfort through several surgical stages [21].

Furthermore, the pain and difficulty during hygienization of the prosthesis could lead to plaque accumulation around the peri-implant tissues [22, 23] and cause discomfort to the patient and mucosal inflammation. An adequate width of keratinized tissue around implants could provide a prosthetic favorable environment, facilitate precise prosthetic procedures, and allow adequate oral hygiene maintenance by the patient, which would help to prevent gingival recession [5]. In addition, wider zones of KM can offer more resistance to the forces of mastication and frictional contact that occur during oral hygiene procedures [15].

One limitation of this technique is that it involves two surgical sites, causing morbidity in both. However, with adequate medication, stabilization of the surgical cement, obtained in this case by the use of both prostheses, and a good follow-up during the first 15 days of healing, we can minimize this limitation. In addition, some percentage of shrinkage should be expected and periodical controls must be performed [19, 24].

In this clinical case report, the patient experienced discomfort, restriction during oral hygiene performance, and plaque accumulation after 30 days of use of the final prosthesis due to a lack of KM, requiring a free gingival graft. The free gingival graft, which was performed after placing of the final prosthesis, allowed the stability of the surgical cement, protection of recipient bed, and immovability of the graft and reestablished physiological functions once the patient was able to continue wearing the prosthesis. Considering the patient's age and health, the use of a free gingival graft was considered a viable and satisfactory treatment option with good outcomes during a 6- and 12-month period of follow-up.

## 4. Conclusion

The free gingival graft, after placing of the final prosthesis and diagnosis of pain and difficulty during hygienization of mandibular prosthesis, was effective in increasing the width of mandibular keratinized mucosa on the buccal side of the interforaminal area and provided an improvement in maintaining the health of peri-implant tissues, which allows for better oral hygiene.
